# Biochemical, Ameliorative and Cytotoxic Effects of Newly Synthesized Curcumin Microemulsions: Evidence from In Vitro and In Vivo Studies

**DOI:** 10.3390/nano11030817

**Published:** 2021-03-23

**Authors:** Abbas Rahdar, Mohammad Reza Hajinezhad, Saman Sargazi, Maryam Zaboli, Mahmood Barani, Francesco Baino, Muhammad Bilal, Esmael Sanchooli

**Affiliations:** 1 Department of Physics, University of Zabol, P.O. Box. 98613-35856, Zabol, Iran; a.rahdar@uoz.ac.ir; 2 Basic Veterinary Science Department, Veterinary Faculty, University of Zabol, P.O. Box. 98613-35856, Zabol, Iran; hajinezhad@uoz.ac.ir; 3 Cellular and Molecular Research Center, Resistant Tuberculosis Institute, Zahedan University of Medical Sciences, Zahedan 98167-43463, Iran; 4 Department of Chemistry, University of Birjand, Birjand 97174-34765, Iran; Zaboli_maryam@yahoo.com; 5 Department of Chemistry, Shahid Bahonar University of Kerman, Kerman 76169-14111, Iran; mahmoodbarani7@gmail.com; 6 Institute of Materials Physics and Engineering, Applied Science and Technology Department, Politecnico di Torino, Corso Duca degli Abruzzi 24, 10129 Torino, Italy; 7 School of Life Science and Food Engineering, Huaiyin Institute of Technology, Huaian 223003, China; bilaluaf@hotmail.com; 8 Department of Chemistry, University of Zabol, P.O. Box. 98613-35856, Zabol, PIran; esmael.sanchooli@uoz.ac.ir

**Keywords:** biomaterials, nanocarrier, oil-in-water F127 microemulsions, curcumin, hepatoprotective, antioxidant, nephrotoxicity

## Abstract

Curcumin is known to exhibit antioxidant and tissue-healing properties and has recently attracted the attention of the biomedical community for potential use in advanced therapies. This work reports the formulation and characterization of oil-in-water F127 microemulsions to enhance the bioavailability of curcumin Microemulsions showed a high encapsulation efficiency and prolonged release. To investigate the interactions of curcumin with one unit of the polymeric chain of surfactant F127, ethyl butyrate, and sodium octanoate, as well as the interaction between ethyl butyrate and one unit of the F127 polymer chain, the Density Functional Theory (DFT) calculations at the M06-2X level of theory, were performed in water solution. The MTT assay was used to assess the cytotoxicity of free and encapsulated curcumin on non-malignant and malignant cell lines. Combination effects were calculated according to Chou-Talalay’s principles. Results of in vitro studies indicated that MCF7 and HepG2 cells were more sensitive to curcumin microemulsions. Moreover, a synergistic relationship was observed between curcumin microemulsions and cisplatin in all affected fractions of MCF7 and HepG2 cells (CI < 0.9). For in vivo investigation, thioacetamide-intoxicated rats received thioacetamide (100 mg/kg Sc) followed by curcumin microemulsions (30 mg/kg Ip). Thioacetamide-intoxicated rats showed elevated serum liver enzymes, blood urea nitrogen (BUN), and creatinine levels, and a significant reduction in liver superoxide dismutase (SOD) and catalase (CAT) activities (*p* < 0.05). Curcumin microemulsions reduced liver enzymes and serum creatinine and increased the activity of antioxidant enzymes in thioacetamide-treated rats in comparison to the untreated thioacetamide-intoxicated group. Histopathological investigations confirmed the biochemical findings. Overall, the current results showed the desirable hepatoprotective, nephroprotective, and anti-cancer effects of curcumin microemulsions.

## 1. Introduction

Curcumin, obtained from *Curcuma longa* rhizomes, has displayed a wide range of immunomodulatory, antioxidant, and anti-inflammatory activities. Early reports have revealed the promising protecting effects of curcumin against oxidative stress-mediated renal dysfunction, liver injury, cardiotoxicity, neuropathy, and immune dysfunction [1]. However, several physicochemical disadvantages limit the therapeutic efficacy of curcumin, including its chemical instability, photo-degradation, fast precipitation in water-based solvents, short half-life, and rapid metabolism, leading to poor bioavailability. Previous reports have shown that new nano-delivery systems provide effective solutions for overcoming the pharmaceutical drawbacks of curcumin, leading to significant therapeutic efficacy enhancement [2,3,4,5,6,7,8].

Curcumin was reported to enhance the sensitivity of resistant MCF-7 cells to conventional anti-cancer agents. As a yellow pigment from turmeric, the phenolic content of curcumin triggers apoptotic cell death through mitochondrial hyperpolarization in human hepatoma cells [9]. It has been widely utilized in blending with alkylating agents to enhance the anti-cancer efficacy against different tumor cells [10,11].

Nowadays, nanotechnology is emerging as an exciting field that finds broad-spectrum applications in nutrition, cosmetics, and biomedical products to improve the bioavailability of therapeutic molecules or drugs [3,4,6,8,12,13,14,15,16,17,18,19,20,21,22,23]. In contrast to larger-scale counterparts, nanoscale materials often display more appealing physical and chemical attributes as well as attractive biological potentialities [19,24,25,26,27,28,29]. The bioavailability could be markedly enhanced by the increased dissolution rate because of the reduced particle size of the active pharmaceutical constituent [30]. Therefore, this approach might be considered a practical solution to enhance the bioavailability and solubility of poorly soluble drug molecules.

Among the various colloidal systems with potential solubilizing applications for hydrophobic compounds, oil-in-water emulsions represent one of the most technically-appealing alternatives for such purposes [31]. Emulsions are categorized into microemulsions, nanoemulsions, and macroemulsions. As anticipated from their names, this classification is based on the size of the dispersed phase particles. Still, the significant difference among these types of emulsions is thermodynamic stability [32]. Microemulsions are recognized to be the preferred candidates in the development of solubilization platforms owing to their high thermodynamic stability and the requirement of minimum mechanical energy for their fabrication. Furthermore, their substantial specific surface area makes them suitable vehicles for promoting the availability of solubilized substances [33,34].

Several reports have demonstrated the potential utilization of microemulsions as a delivery platform to improve the therapeutic action of targeted molecules, thus diminishing the toxicity of the drug to humans [13,15,17,23,35,36,37,38,39,40,41,42,43,44,45,46]. Dhumal et al. [47] developed a new self-micro-emulsifying drug delivery system (SMEDDS) by using oleic acid (semi-synthetic)-originated bicephalous heterolipid (E1E) for enhancing the bioavailability and solubility of curcumin. The curcumin solubility in E1E was 2.6- and 14-folds increased compared to using ethyl oleate and oleic acid, respectively. The E1E-based SMEDDS showed an excellent curcumin encapsulation efficacy of 70.52 mg g^−1^ and formed a spontaneous microemulsion by inclusion into aqueous phase with a polydispersity index and average globule size of 0.243 and 22.39 nm, respectively. After the delivery via E1E-based SMEDDS, the absorptive ability of curcumin was improved to 26 folds. A freeze-drying method was used to prepare novel curcumin-loaded hydroxypropylmethylcellulose (HPMC)-based sponge formulations with a mean pore size ranging from 43.36 to 123.22 nm. The optimal sponge formulation gave rise to an average microemulsion diameter of 34.80 nm, leading to a complete release of curcumin within 2 h. After oral administration of curcumin-loaded sponges in rabbits, the area under the concentration-time curve (AUC) was 5- to 7-folds increased as compared with the normal curcumin powder [48]. In order to determine the potential protective effects of curcumin microemulsions against the hepatotoxicant thioacetamide, rats received three injections of thioacetamide. Thioacetamide administration is an excellent model to induce sub-acute renal and hepatic injury and oxidative stress in laboratory animals. It is worth noting that the concentration of oil, drug, surfactants, and co-surfactants might influence the stability of drug/biomolecule-encapsulating oil-in-water microemulsion systems. Given their low toxicity and great stability, Pluronics have gained importance as preferred non-ionic surfactants in designing oil-in-water microemulsions for pharmaceutical purposes rather than ionic surfactants [49,50,51,52,53]. Our research group recently developed Pluronic F127-based biodegradable and biocompatible microemulsions to increase the solubility and delivery of tocopherol. Besides the high encapsulation capacity, the as-formulated micro emulsion-based nanocarriers presented a sustained release profile of the cargo [39]. Nevertheless, no report has been conducted so far on preparing oil-in-water F127 microemulsions to augment the bioavailability of curcumin. The effect of intraperitoneal injections of curcumin microemulsions on thioacetamide-induced oxidative stress has also not been examined yet. Therefore, this study investigates the nanoformulation and characterization of such oil-in-water Pluronic F127 microemulsions. The biochemical, ameliorative, and cytotoxic effects of curcumin microemulsions were specifically evaluated to provide evidence for in vitro and in vivo application of this novel formulation.

## 2. Materials and Methods

### 2.1. Materials

Standard laboratory grade chemicals, including curcumin, sodium octanoate (commonly called sodium caprylate), and ethyl butyrate were provided by Sigma Chemical Co. (Taufkirchen, Germany). Pluronic surfactant F127 was procured from BASF Inc. (Mount Olive, NJ, USA) DMSO, 3-(4,5-dimethylthiazol-2-yl)-2,5-diphenyl-tetrazolium bromide (MTT), and cisplatin were procured from Sigma-Aldrich Chemical Company (St Louis, MO, USA). Fetal bovine serum (FBS) was procured from Biochrome (Berlin, Germany). Culture mediums, including Dulbecco’s Modified Eagle’s medium (DMEM) and RPMI, and also antibiotic/antimycotic solution were supplied by INOCLON (G. Innovative Biotech Co, Tehran, Iran). Plastic materials were obtained from SPL life Science (SPL, Seoul, Korea). All other chemicals/reagents were of a high quality and used as such.

### 2.2. Formulation of Curcumin-Incorporated Microemulsions

**Scheme 1:**
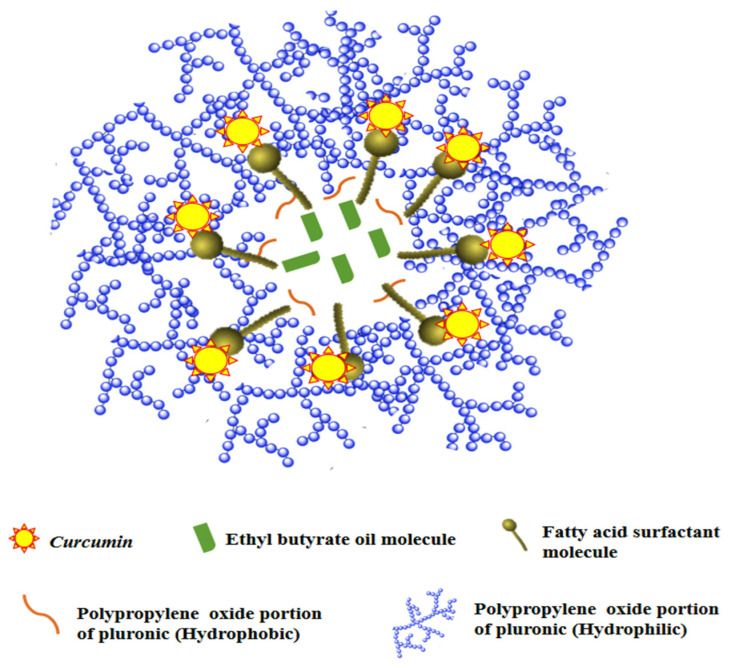
Computational study.

The synthesis of curcumin-incorporated oil-in-water microemulsions [54] involved the vigorous stirring of a suitable amount of sodium caprylate, F127, PBS (pH = 7.4) at a fixed curcumin-to-Pluronic molar ratio of 0.043. Scheme 1 portrays the schematic representation of the newly synthesized Pluronic microemulsion structure and contents.

### 2.3. Computational Study

The geometry of monomers and the obtained complexes were optimized by using DFT at M06-2X [55] functional level. The M06-2X functional is able to perform both electrostatic and dispersion interactions. All calculations were performed by applying the standard 6-31G* basis set within the Gaussian 03 program package [56]. The solvation (water) effect was calculated by using the polarizable continuum model (PCM) of the self-consistent reaction field (SCRF) [57,58]. The interaction energy (E_int_) values were obtained by subtracting the energies of the isolated monomers from the energy of the complex:

(1) $$E_{int}=E_{\mathrm{complex}}-E_{monomer_{1}}-E_{monomer_{2}}+E_{\mathrm{BSSE}}$$

where $E_{\mathrm{complex}}$, $E_{monomer_{1}}$, and $E_{monomer_{2}}$ denote the total energy of obtained complexes after interaction of two monomers and the total energies of the optimized monomers, respectively. The E_BSSE_ value shows the basis set superposition error (BSSE) that was obtained by applying the counterpoise procedure of Boys and Bernardi according to the below Equation [59]:

(2) $$E_{\mathrm{BSSE}}=\;E{\left({monomer_{1}}^{\prime }\right)}_{monomer_{1}\;}\pm \;E{\left(monome{r_{1}}^{\prime }\right)}_{\mathrm{complex}\;}+\;E{\left(monome{r_{2}}^{\prime }\right)}_{monomer_{2}\;}\pm \;E{\left(monome{r_{2}}^{\prime }\right)}_{\mathrm{complex}\;}$$

where $E{\left(monome{r_{1}}^{\prime }\right)}_{complex\;}$ shows the energy calculated for *monomer*_1_ with its geometry in the complex and the complete set of basic functions applied to describe the dimer. Moreover, $E{\left(monome{r_{1}}^{\prime }\right)}_{monomer_{1}\;}$ is the energy structure of *monomer*_1_ on the complex calculated by using its corresponding basis set [60]. The Atoms in Molecules (AIM) method was used to analyze the topological parameters related to the intermolecular hydrogen bond (H-bond) formation [61,62]. The AIM analysis was carried out by using the AIM2000 program [63] at the M06-2X/6-31G* level of theory. The natural bond orbital (NBO) method [64,65] was used to analyze the natural population and the charge transfer during the reaction course.

### 2.4. Characterization of Curcumin-Loaded Microemulsions by Dynamic Light Scattering (DLS)

DLS characterization of curcumin-incorporated microemulsions was carried out by using an ALV-5000F Goniometer System coupled with a diode-pumped solid-state laser to supply polarized incident light. This technique is commonly used to assess the size of nanomaterials and nano-systems [66,67,68,69]. The measurement equipment was also integrated with a digital correlator (ALV SP-86) with a sample range of 25 ns to 100 ms. DLS was performed at an angle of θ = 90° to the incident ray by calibrating the intensity scale by toluene against scattering. Before measuring, the sample solutions were directly filtered into scattering cells using Millipore Millex filters (0.22 μm porosity) and equilibrated for 10 min at the required temperature. In order to acquire a fitted correlation function, the sampling time was 5–10 min. All the experiments were carried out three times.

### 2.5. Entrapment Efficiency of Curcumin

In order to calculate the content of curcumin in the formulations, the UV-spectrophotometric approach was used (Agilent Technologies, Cary 50, Santa Clara, California, USA) [8,17,23]. Curcumin stock solution (30 μg/mL) was diluted with ethanol/PBS 7.4 (1:1) from the curcumin commercial product. At wavelengths of 200 to 700 nm, the absorbance peak of curcumin was initially determined. The curcumin showed a characteristic peak at a wavelength of 420 nm. For calibration curves, working standard solutions of curcumin were prepared by diluting the stock solution within a concentration range of 30–0.25 μg/mL, and spectrophotometric determination was carried out at 420 nm. The curve (R^2^ = 0.9895) was found to be linear and reproducible. Curcumin-containing microemulsions were then centrifuged at 20,000 rpm for 60 min (model MC-20000, Medline, UK). The curcumin content of the resulting supernatant solution was calculated by absorbance measurement at 420 nm. Eventually, the encapsulation efficiency (EE percent) was determined as the difference between the total microemulsion curcumin (Crcm) content and the free supernatant curcumin according to Equation (3):

(3) $$EE\;\left(\%\right)=\frac{\left(Total\;Crcm-Free\;Crcm\right)}{Total\;Crcm}\times 100$$

### 2.6. Release Study

Release activity was tested using a dialysis technique with a 6000 Da pore size dialysis membrane [4,8,17]. For at least 12 h prior to use, the dialysis bag was immersed in the PBS buffer as a receptor. As a donor portion, 1 mL of curcumin solution or curcumin-loaded microemulsion was placed in the dialysis bag. In the receiver chamber, 50 mL PBS 7.4/ethanol was added. Curcumin release tests were conducted over 24 h at 37 °C and a speed of 90 rpm; 1 mL of the buffer medium as the receiver was collected at various time intervals, and subsequently, the same quantity of fresh buffer (preheated at 37 °C before replacement) was added to a receiver to maintain a steady volume. The UV spectrophotometer measured the absorbance of the samples at a 420 nm wavelength. The released curcumin was withdrawn and analyzed in a quartz cuvette with an area of 1 × 1 cm^2^ by using a UV-Vis spectrophotometer (Agilent Technologies, Cary 100, Santa Clara, CA, USA). Each experiment was measured in triplicate.

By fitting the release results to a zero-order, first-order, Higuchi and Korsmeyer-Peppas models, the release kinetics of curcumin were predicted [8]. The profile of percent release vs. time for the zero-order, the profile of log of percent release vs. time for the first-order, the profile of the percent release vs. the square root of time for the Higuchi model, and the profile of log of percent release vs. log of time for Korsmeyer-Peppas model was plotted.

### 2.7. In Vitro Studies

#### 2.7.1. Cell Lines and Cultivation Conditions

Human umbilical vein endothelial cells (HUVECs) were a kind gift from Dr. Roghayeh Sheervalilou. HUVECs were cultured in DMEM medium. HEK293 cells, as another non-malignant cell lines derived from the human embryonic kidney, were procured from the cell collection of Royan Institute (Tehran, Iran) and grown in high-glucose DMEM. MCF-7 human breast cancer and HepG2 human hepatoma cell lines were procured from the Cell Repository of the Research Institute of Biotechnology, Ferdowsi University of Mashhad, Iran, and were cultivated in RPMI-1640 culture media. Both cell lines tested negative for mycoplasma contamination. For cultivation of all cell lines, the culture medium was supplemented with 10% heat-activated FBS, penicillin (950 U/mL), amphotericin B (250 µg/mL), and streptomycin (50 µg/mL), and was maintained at standard cell culture conditions [70].

#### 2.7.2. MTT Cytotoxic Assay

Cytotoxicity exerted by free curcumin and curcumin-loaded microemulsions on cell lines was investigated by performing the MTT assay [71]. Cells (5 × 10^3^ cells/well) were seeded in 96-well microplate in triplicates, incubated overnight, and treated with free curcumin and curcumin-loaded microemulsions from 0 to 300 µg/mL. Cisplatin was added in the range of 0 to 32 µg/mL concentrations for comparing its cytotoxic activity with free and encapsulated curcumin. After incubation for 48 h, 20 μL of MTT reagent (5 mg/mL) was included in each well following incubation at 37 °C for 4 h. Next, the culture medium was cautiously removed and replaced with the addition of 200 μL of DMSO for complete solubilization of formazan crystals. The absorbance was read at 570 nm by using a Gemini microtiter plate reader. The viable cells percentage was assessed as the ratio of sample OD to the control OD. The results represent the mean ± SD of three independent experiments. GraphPad Prism software version 7.0 was used to measure the half-maximal inhibitory concentration (IC_50_) as µg/mL.

#### 2.7.3. Analysis of Combined Drug Effects

Analysis of drug interactions was performed by using Chou and Talalay’s methods [72]. For this purpose, HepG2 and MCF7 cells were simultaneously treated with free or encapsulated curcumin and cisplatin as a single agent in fixed-ratio combinations. Increasing concentrations, each diluted 1:2 within the acceptable ranges, were used. For each level of fraction affected (Fa), the combination index (CI) for indicating drug interactions was calculated via CompuSyn software (Version 1.0), where CI  <  1, CI  =  1, and CI  >  1 represents synergism, additivity, and antagonism, respectively.

### 2.8. Animal Treatments and Grouping Design

The experimental works on laboratory animals were conducted in the laboratory animal center of the University of Zabol, Zabol, Iran. In the current work, thirty-two male adult white rats (mean weight 223 g) were housed in poly (carbonate) cages. The laboratory animal house which the animals held had 25 °C with a 12 h light/12 h dark program. Animals had free access to standard rodent chow pellets (manufactured by Javaneh-Khorasan company, Mashhad, Iran) and sterile tap water. In order to acclimatize the rats for the experimental procedure, animals were kept at a two-week adaptation period before the experiments. The experimental methods and rat handling were performed according to the international ethical procedures for the use and care of laboratory rodents (from NIH Publication No.85-23). The experimental procedure was performed according to guidelines of the ethical research committee of the University of Zabol, Zabol, Iran (IR.UOZ.REC.1399).

Animals were allocated randomly into four groups, as follows. The healthy control rats received 0.5 mL intraperitoneal injection of saline for 28 days. The rats of the second group were daily treated with intraperitoneal injections of curcumin microemulsions at a dose of 30 mg/kg for 28 days. The dose of curcumin microemulsions was selected according to previous reports [73] and our preliminary experiments.

The third group was treated by three consecutive subcutaneous injections of thioacetamide (100 mg/kg) for four weeks. The fourth group received three successive subcutaneous injections of thioacetamide (100 mg/kg) at 24 h intervals following 30 mg/kg curcumin microemulsions for four weeks. The doses of thioacetamide were selected based on previous studies and preliminary experiments [74]. After four weeks of treatments, blood samples were collected by the retro-orbital sinus puncture method. The obtained blood samples were immediately sent to the clinical pathology laboratory of the University of Zabol for determining biochemical parameters. After immediate centrifugation of blood samples (3000 rpm for 5 min), the obtained serum samples were separated from blood and preserved at −20 °C.

### 2.9. Serum Biochemical analysis

Serum ALT, AST, blood urea nitrogen (BUN), and creatinine were determined by using Pars Azmoon kits (Tehran, Iran), according to the manufacturer’s instructions. Analysis of biochemical parameters was done by Selectra Pro M autoanalyzer. Serum malondialdehyde content was measured by adopting the method described by Ohkawa et al., with minor modifications [75]. Serum catalase activities were determined by using the Goth method [76]. Furthermore, the SOD activity was measured by the Sun method with minor changes [77].

### 2.10. Statistical Analysis

Serum biochemical parameters were studied by one-way ANOVA followed by Tukey’s post-hoc test. The biochemical results were examined using the SPSS software (version 20.0), where significant differences among the groups were set at *p* ˂ 0.05.

## 3. Results and Discussion

### 3.1. Synthesis and Characterization of Curcumin-Loaded Microemulsions

**Figure 1:**
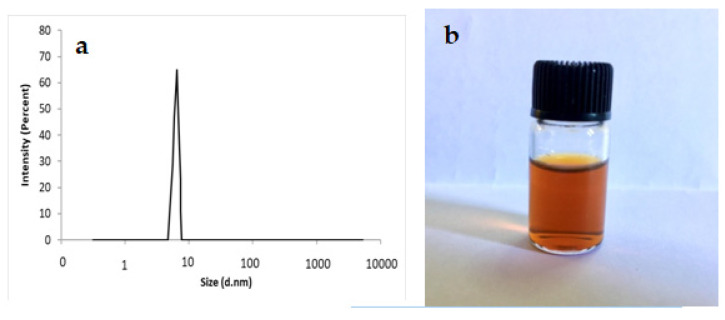
DLS results: (**a**) size distribution; (**b**) visual stability of microemulsion after six months.

Figure 1a shows a scheme of size distribution for as-prepared microemulsions. The size of curcumin-based microemulsions measured to be approximately 8 nm.

Regarding stability of microemulsions, the PDI obtained from DLS analysis of microemulsions showed values (0.1–0.2), indicating a size homogeneity of the droplets in the total microemulsion. The stability of the microemulsion was confirmed visually after six months of preparation and no aggregation was observed (Figure 1b).

### 3.2. Quantum Mechanics Calculations

The interactions of curcumin with one unit of the polymeric chain of surfactant F127, ethyl butyrate, and sodium octanoate were studied; furthermore, the interaction between ethyl butyrate with one unit of F127 polymer chain was investigated in water solution as well. The obtained complexes have been introduced with CURF, CUREthyl, CURSodium, and EthylF, respectively. The geometry optimization of monomers and the obtained complexes was done at the M06-2X level of theory. The optimized structure of monomers and the resulting complexes are shown in Figures S1 and S2 in the supplemental material. All possible positions for interaction between the mentioned monomers were considered and indicated in Figure S2.

Moreover, the adsorption energy values are indicated in Figure S2, which shows that the most stable complexes belong to the interaction between curcumin and F127, i.e., CURF1 and CURF2 complexes.

#### 3.2.1. AIM Analysis

The topological properties of electron densities are generally interpreted according to the quantum theory of atoms in molecules. AIM theory is a convenient method in quantum mechanics to examine various interactions [78]. Topological parameters are often used to detect the presence of hydrogen bond interactions and are also applied as descriptors to describe the H-bond strength [79,80]. The electron density, Laplacian of electron density, and total energy density values (*ρ*(r), ∇^2^*ρ*(r), H(r), respectively) at the bond critical points (BCPs) of all complexes are indicated in Table S1. The results of NBO analysis are reported in Table S2.

The molecular graph of one of the most stable complexes is displayed in Figure S3, which demonstrates distinctly the bond critical point (red balls), ring critical point (yellow balls), and the bond paths.

The Espinosa method was applied as a powerful technique for estimating H-bond energy [81]. In this method, the individual hydrogen bond energies ($E_{HB}^{*})\;$ were estimated using the electron densities at the hydrogen bond critical points. The maximum electron density and $E_{HB}^{*}$ values at the H-bond critical points belong to CURF2 and CURSodium3 complexes at the H_47_-O_54_, H_66_-O_9_ bonds, respectively (see Table S1). There is a good correlation between the $E_{HB}^{*}$ values and the geometrical parameters. Therefore, it can be concluded that the geometrical parameters are good descriptors to show the H-bond strength. It is determined that the shorter the H···Y (Y is the proton acceptor), the stronger the H-bond. Therefore, it is reasonable that the H_47_-O_54_ in the CURF2 complex and the H_66_-O_9_ bond in the CURSodium3 complex have a minimum bond length (see Figure S4).

The positive ∇^2^*ρ*(r) and H(r) values at the contact points indicate the weak H-bonds between the studied monomers except for H_47_-O_54_ contact of CURF2 complex and H_66_-O_9_ bond in CURSodium3, since ∇^2^*ρ*(r) > 0 and H(r) < 0 show medium-strength intermolecular interaction with partially covalent nature in these points. The *ρ*(r) at BCP of X···H contact are well correlated with the $E_{HB}^{*}$ value; consequently, a higher value of *ρ*(r) corresponds to stronger H-bond interactions. The correlation between ∇^2^*ρ*(r) and $E_{HB}^{*}$ at intermolecular interaction is demonstrated in Figure S5.

#### 3.2.2. HOMO–LUMO Analyses

The LUMO indicates the ability to acquire an electron and act as an electron acceptor, while the HOMO shows the ability to donate an electron. The energy gap (E_g_) and the HOMO and LUMO orbitals for the most stable complex (CURF2) are shown in Figure S6.

The HOMO and LUMO energy values of monomers were applied to calculate the |${\mathrm{HOMO}}_{(monomer_{1}}$_)_-$\;{\mathrm{LUMO}}_{(monomer_{2}}$_)_| and the |${\mathrm{HOMO}}_{\left(monomer_{2}\right)}$-${\mathrm{LUMO}}_{\left(monomer_{1}\right)}$| values and the results were listed in Table S3.

#### 3.2.3. Thermodynamic Parameters

The thermodynamic parameters of all complexes, including Gibbs free energy (ΔG), enthalpy (ΔH), and entropy (ΔS) were calculated at the standard situation (STP, 1 atm, and 298 K). The obtained results are listed in Table S4.

### 3.3. Entrapment Efficiency

One of the significant physiochemical properties in the design of drug nanostructures is encapsulation efficiency (EE) [5,8,12,82]. High EE guarantees treatment efficacy provided by loaded substances at a lower dose than that needed for free chemical molecules to be administered, thus minimizing the level of adverse side effects [3,4,5]. The curcumin microemulsion encapsulating performance was 88 ± 1.5%. Sharma et al. reported an EE of 69.98 ± 0.21 to 83.32 ± 0.15% for curcumin-loaded Pluronic F-127 microemulsions [83]. Chen et al. prepared curcuminoid microemulsions consisting of soybean oil, Tween 80, ethanol, and water with an EE of 85.7% [84]. Based on this study, this high EE percentage may be due to interactions between curcumin and microemulsions, which can result in a more rigid microemulsion membrane and affect curcumin release. Furthermore, the microemulsion core is hydrophobic, and it can be said that curcumin is mainly encapsulated in the core of the microemulsion.

### 3.4. In Vitro Release Experiment

**Figure 2:**
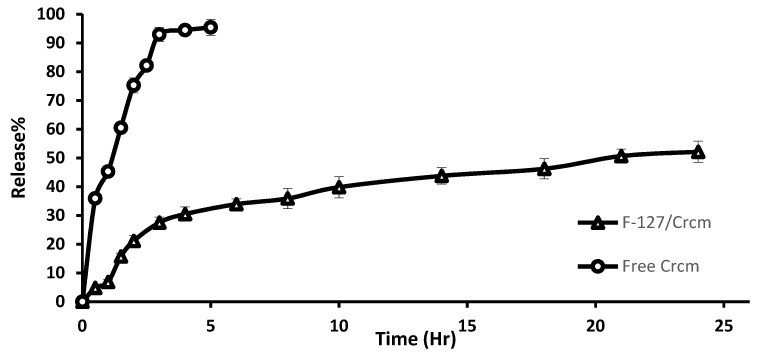
In vitro release of free curcumin (Crcm) and curcumin-loaded microemulsion (F-127/Crcm) at PBS 7.4/ethanol (1:1) and 37 °C after 24 h.

The curcumin solution and curcumin-loaded microemulsion in vitro release experiments were performed using dialysis methods at PBS 7.4/ethanol (1:1) and 37 °C. The free curcumin (Crcm) release rate was significantly faster (like a burst-release behavior) than the curcumin-loaded microemulsion, as shown in Figure 2. Curcumin release reached only 52% after 24 h for curcumin-loaded microemulsion, which showed a slow release rate. This is a significant achievement potentially enabling a prolonged therapeutic effect. In a previous study, Sharma et al. reported that curcumin release of about 74% in 8 h for optimized microemulsion formulations [83].

**Figure 3:**
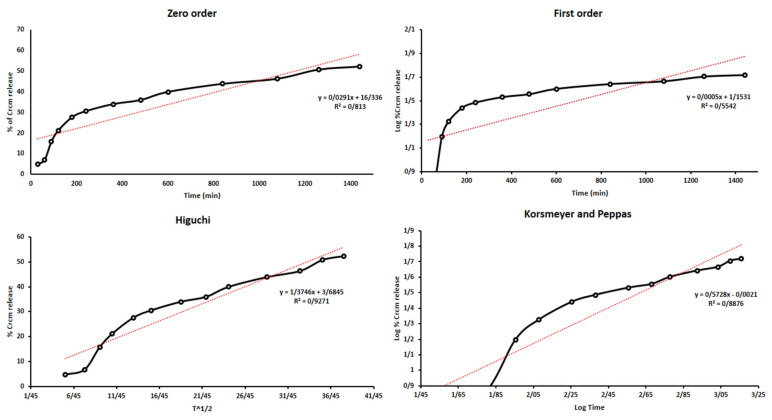
Profiles of different kinetic modes for release of curcumin from microemulsion; the black line is fitting mode of different kinetic release models, and red line is linear fitting.

By matching the first-order, zero-order, Higuchi, and Korsmeyer-Peppas models, curcumin release kinetics were evaluated. As shown in Figure 3, the Higuchi model was better suited to the microemulsion curcumin release rate (R^2^ = 0.9271). This kind of release describes the drug release as a diffusion process based on Fick’s law, which is square root time-dependent. At the beginning of the process and in the presence of water, the microemulsion may gradually expand, allowing the loaded drug to spread through the membrane. The lipophilic fatty cores, however, will decrease the diffusion coefficient of curcumin [8].

The effect of different parameters on the release of active ingredients from nanocarriers has been evaluated in several studies [85,86,87]. Mikesh et al. prepared curcumin microemulsion by water titration approach by biocompatible components for intranasal delivery [88]. In their in vitro release analysis, mucoadhesive microemulsion was found to show more prolonged release. Kinetic models have been fitted with release data showing that Fickian is the release model.

### 3.5. In Vitro Assessments

**Figure 4:**
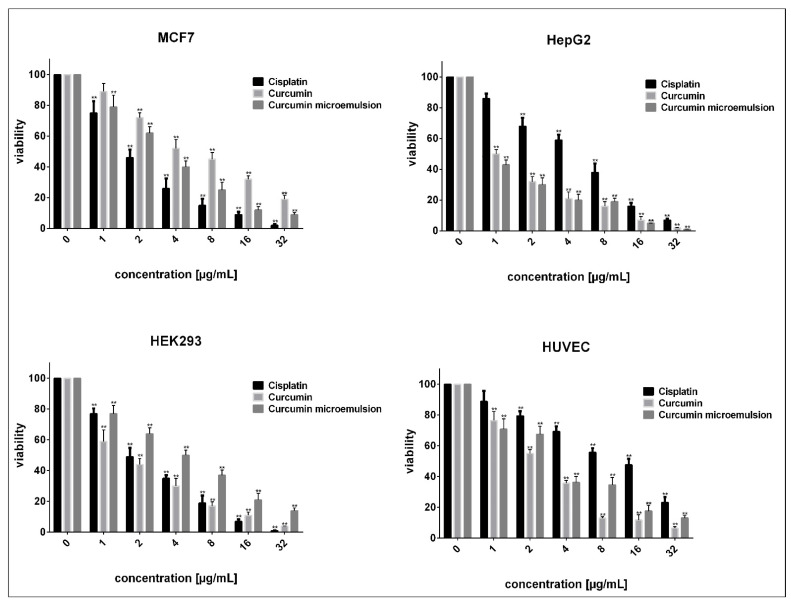
Cytotoxic effects of free curcumin, curcumin microemulsion, and cisplatin (as positive control) on malignant (HepG2 and MCF7) and non-malignant (HUVEC and HEK293) cell lines using MTT assay. The results represent the mean ± SD of three independent experiments. (** *p* < 0.05 compared with untreated cells).

Both free curcumin and curcumin microemulsions diminished the viability of non-malignant and malignant cells in a concentration-dependent manner (Figure 4). Within the described ranges, the IC_50_ values of free curcumin and curcumin microemulsions were, respectively, 55.75 µg/mL and 27.46 µg/mL in MCF7 cells, 9.61 µg/mL and 8.24 µg/mL in HepG2 cells, 21.55 µg/mL and 30.24 µg/mL in HUVECs, and 14.79 µg/mL and 37.83 µg/mL in HEK293 cells. Compared to free curcumin, malignant cells were more sensitive to newly synthesized curcumin microemulsions (lower IC_50_). In comparison, the normal cells derived from human embryonic kidney and umbilical vein tissues were more resistant to encapsulated curcumins (higher IC_50_). However, in free and microemulsion formulation, curcumin induced much less toxicity than cisplatin (higher IC_50_ on both malignant and non-malignant cell lines). This indicates that cisplatin can enhance cancer cell killing much more effectively than newly developed curcumin microemulsions.

**Figure 5:**
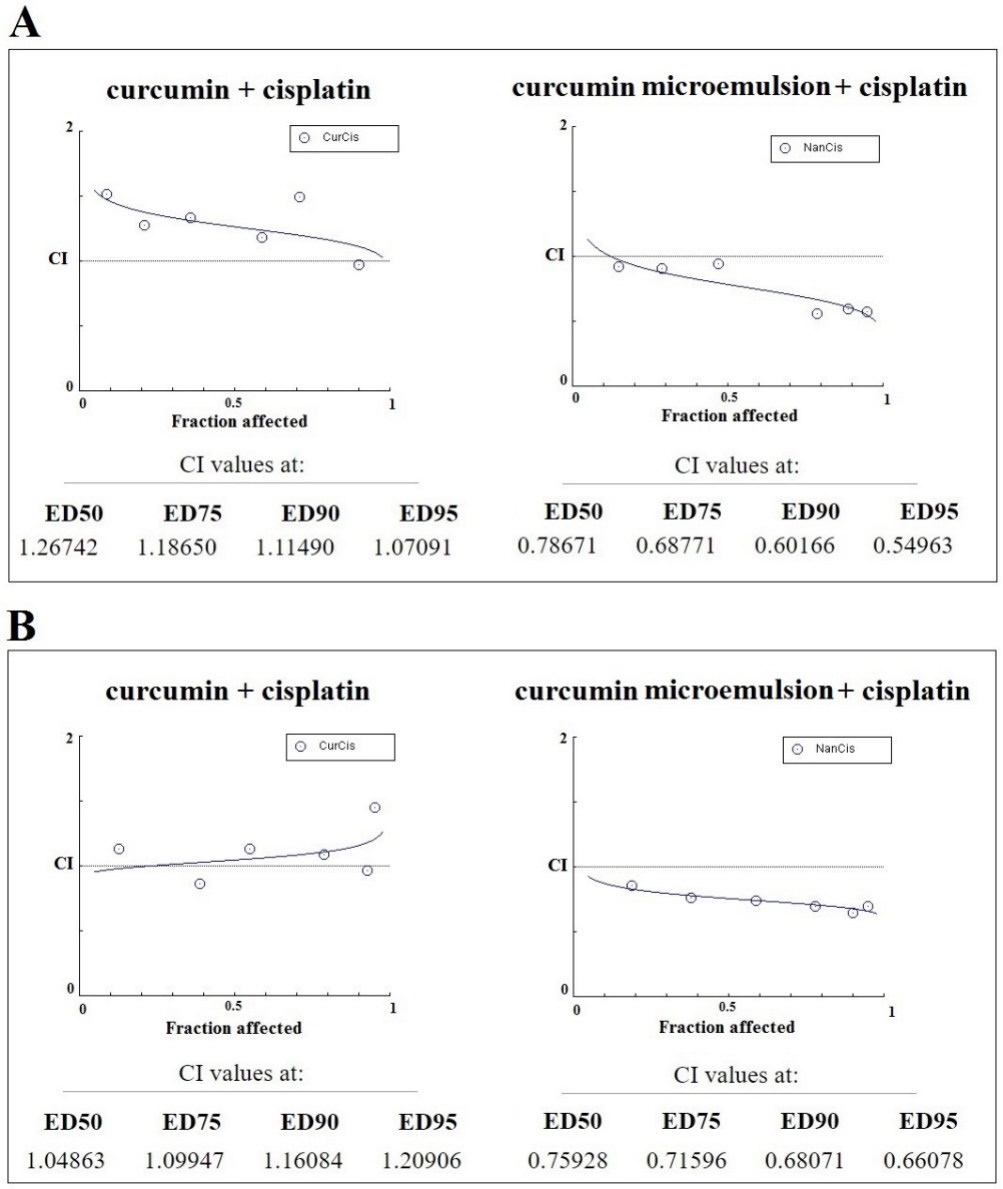
Analysis of drug interaction between free or encapsulated curcumin and cisplatin in (**A**) HepG2 and (**B**) MCF7 cancer cells in different effective doses (EDs).

Later, we aimed to assess the interaction between cisplatin, a conventional alkylating agent, and free/encapsulated curcumin in malignant cells. Results of combination analysis revealed that a combination of free curcumin and cisplatin mediated additive or mild antagonistic effects on HepG2 (Figure 5A) and MCF7 (Figure 5B) cells (CI > 1). Interestingly, we observed a synergistic relationship between curcumin microemulsions and cisplatin in all affected fractions of HepG2 (Figure 5A) and MCF7 (Figure 5B) cells (CI < 1). These findings suggest that the newly developed curcumin microemulsions enhance cisplatin cytotoxicity in cells derived from liver or breast tissues of cancer patients.

Combination therapy enhances the efficacy of anti-cancer drugs compared with the mono-therapy approach and reduces drug resistance or adverse effects [89,90]. Cisplatin, an alkylating agent widely used in treating different cancers, induced putative toxicity and is the principal cause of nephrotoxicity, hepatotoxicity, ototoxicity, and allergic reactions [91,92]. In this regard, many efforts have been made to exploit the synergy between cisplatin and the anti-cancer drugs with totally different mechanisms of action, including encapsulation of one or two drugs in a newly formulated nanoscale delivery system. We hypothesized that if cisplatin synergizes with another anti-cancer drug, lower concentrations of cisplatin are needed in combination with that agent to achieve similar cytotoxicity to cancer cells. We performed combination drug analysis to investigate if cisplatin synergizes with curcumin microemulsions in cancer cells and found a synergistic relationship between the two drugs, which might be a promising outcome. However, our results did not entirely agree with the findings reported by Notarbartolo et al. [10], who found a synergistic relationship between free curcumin and cisplatin in HA22T/VGH, as a poorly differentiated hepatoma cell line. Still, we observed a promising cell-death-inducing capability of the synthesized microemulsions against malignant liver and breast cells. Compared with free curcumin, nano-curcumin exerted higher cytotoxicity in malignant cells, specifically in MCF7 breast cancer cells. This is because our new formulation increased the solubility of curcumin, which is a slightly soluble drug, and enhanced its delivery to the tumor site. Overall, the incorporation of curcumin in microemulsion increased curcumin cytotoxicity and cisplatin cytotoxicity against cancer cells. Hence, it can be suggested that the synthesized curcumin microemulsions could serve as a versatile and efficient nano-delivery system for curcumin, a drug that practically requires solubilizing agents for delivery.

### 3.6. Biochemical Assessments

Table 1 shows serum biochemical parameters and oxidative stress status in rats. Rats in the control group showed normal serum BUN and creatinine levels, normal liver enzymes, malondialdehyde (MDA), and liver antioxidant enzyme activities. The animals treated with curcumin microemulsions also had healthy liver and kidney function, normal lipid peroxidation level, and enhanced liver antioxidant-enzyme activities. The thioacetamide-treated rats had elevated serum AST and ALT levels in contrast to the control rats (*p* < 0.05 and *p* < 0.01, respectively). Serum BUN and creatinine levels also increased in rats who received the subcutaneous injection of thioacetamide (*p* < 0.001). The thioacetamide-treated rats also had reduced liver catalase and SOD activities (*p* < 0.01 and *p* < 0.05, respectively) with a substantial rise in serum MDA levels (*p* < 0.05). Treatment with curcumin microemulsions restored the elevated levels of BUN, creatinine, AST, and serum MDA to the normal levels. Treatment with curcumin microemulsions also improved liver catalase and superoxide dismutase activities in thioacetamide-treated rats compared to the untreated thioacetamide-intoxicated rats. The current work showed the potential antioxidant and hepatoprotective effects the curcumin microemulsion. The potential antioxidant activity of curcumin microemulsions after oxidative stress induction was investigated in rats. Activities of serum antioxidant enzymes in the liver, serum liver enzymes, kidney function markers, lipid peroxidation, and possible liver toxicity and nephrotoxicity were examined. Four weeks of intraperitoneal injections of curcumin microemulsions caused a significant elevation in liver SOD and catalase activities and a non-significant decrease in liver MDA content.

Previous reports have demonstrated the antioxidant properties of free curcumin and curcumin microemulsions in experimental animals [93,94]. Treatment with curcumin microemulsions also led to a significant hepatoprotective and nephroprotective effect in thioacetamide-intoxicated rats. While numerous studies have inspected the effects of curcumin on liver and kidney function, few works have been conducted on in vivo effects of curcumin microemulsions. In the current work, liver sections showed diffuse hepatocyte ballooning, which is an indicator of reversible liver damage. These histopathological changes were reduced in rats treated with curcumin microemulsions. The decrease in hepatocyte fatty change was accompanied by an improvement in liver and serum antioxidant status. It seems that antioxidant activity is the fundamental mechanism of the hepatoprotective effects of curcumin microemulsions. This confirmed the results of previous studies showing that curcumin microemulsion formulation could increase the hepatoprotective and antioxidant potential of curcumin [95].

The outcomes of the current study revealed that a four-week injection of curcumin microemulsions could reduce the liver histopathological lesions in thioacetamide-treated rats; however, the liver changes were still present, which could be due to the short-term administration of microemulsions. Thioacetamide is an excellent model for inducing liver and kidney damage. By performing in vitro cytotoxic assays, we investigated the cell-death-inducing capability of newly developed curcumin microemulsions compared to free curcumin. Interestingly, in malignant MCF7 and HepG2 cells, curcumin microemulsions induced higher concentration-dependent toxicity than free curcumin. Simultaneously, no reduction in the viability was observed following treatment of normal human cells with curcumin microemulsions, which is a promising result considering that many anti-cancer agents cause undesirable effects, i.e., normal cell death [96].

### 3.7. Histological Results

**Figure 6:**
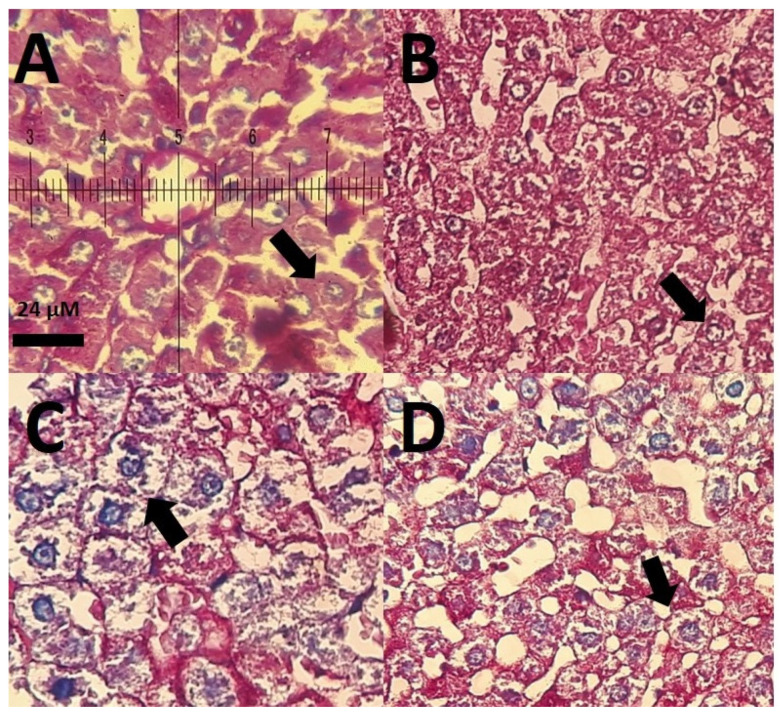
(**A**) Alcian blue-stained section liver section of a control rat showing normal hepatocytes, well-arranged hepatic cords, normal portal triads, and normal central vein (CV). Bar = 24 μm (×40). (**B**) Liver section of a rat received the 30 mg/kg dose of curcumin microemulsions, showing normal morphology of hepatocytes (arrow) and well-arranged hepatic cords. As seen, no histopathological injury is observed. Alcian blue staining (×40). (**C**) liver sections of a rat thioacetamide-treated rat, intensive intra-cytoplasmic fat deposition is observed (arrow); (**D**) liver section of a thioacetamide-intoxicated rat treated with intraperitoneal injections of 30 mg/kg curcumin microemulsions, showing a reduction in cytoplasmic vacuolation (arrow point). Alcian blue staining (40× magnification). Scale bar indicates 40 μm.

**Figure 7:**
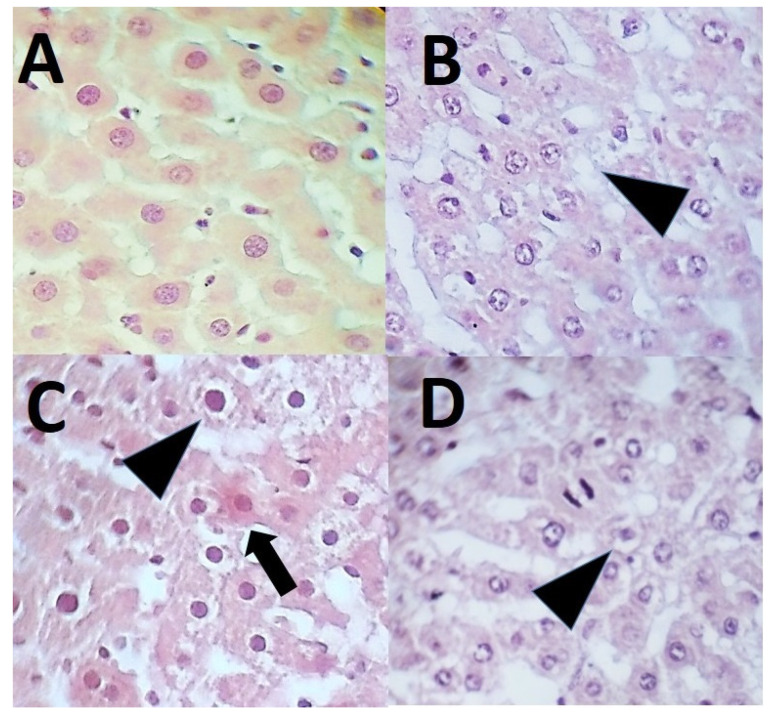
(**A**) Periodic acid-Schiff (PAS) section liver section of a control rat showing liver morphology with normal hepatocytes and hepatic cords. (**B**) The liver section of a rat received the 30 mg/kg dose of curcumin microemulsions, showing normal hepatocytes and hepatic cords, indicating normal histology of the liver. (**C**) liver sections of a thioacetamide-intoxicated rat, intra-cytoplasmic fat vacuoles are observed (arrow); (**D**) liver section of a thioacetamide-intoxicated rat treated with intraperitoneal injections of 30 mg/kg curcumin microemulsions, showing a decrease in fatty change (arrow point). Periodic acid–Schiff (PAS) staining (40× magnification).

**Figure 8:**
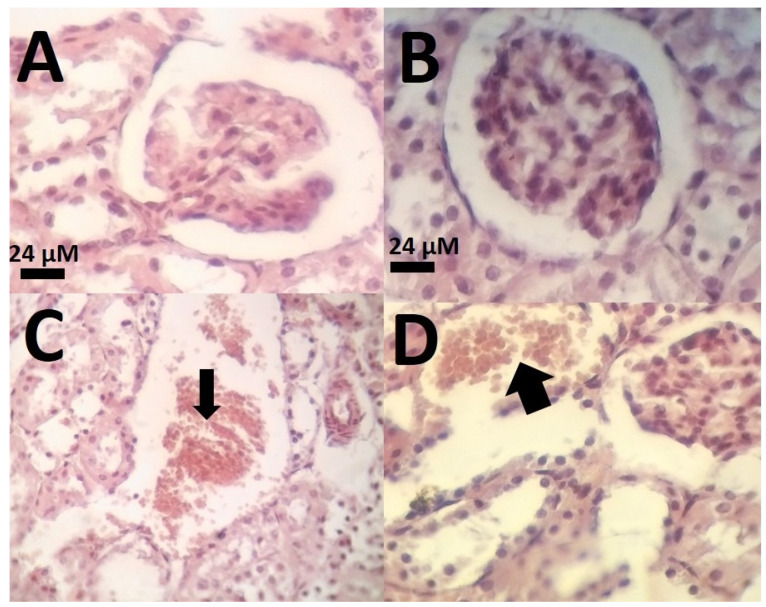
(**A**) Kidney micrograph of a normal control rat, showing normal glomerulus and normal tubules. (**B**): renal micrograph of a rat treated with curcumin microemulsions at a 30 mg/kg dose with normal kidney structure. (**C**): kidney of a thioacetamide-treated rat at a dose of 30 mg/kg; extensive bleeding is present (arrow). (**D**): A kidney PAS-stained section of a thioacetamide-intoxicated rat treated with curcumin microemulsions at a dose of 30 mg/kg shows a reduction in blood congestion in the kidney (arrow). PAS staining. (40× magnification).

The results of histopathological investigation of the liver and kidney are presented in Figure 6, Figure 7 and Figure 8. As shown in Figure 6, control rats had normal histology with well-arranged hepatocytes (Figure 6A). The group that received the curcumin microemulsions also had normal morphology, as seen in Figure 6B. The rats that received intraperitoneal injections of thioacetamide showed perinuclear fat deposition in the cytoplasm (Figure 6C), which were ameliorated in rats receiving the combined administration of thioacetamide and curcumin microemulsions (Figure 6D). Periodic acid-Schiff (PAS) staining of the liver of control rats and curcumin-treated rats was quite normal. As seen in Figure 7A,B, hepatocytes and hepatic cords were quite normal too. In contrast, liver histopathological investigation of thioacetamide-treated rats showed severe fatty change and increasing glycogen storage in the cytoplasm (Figure 7C). In the liver of thioacetamide-intoxicated rats treated with intraperitoneal injections of 30 mg/kg curcumin microemulsion, a decrease in fatty change was observed (Figure 7D).

Kidney micrographs of the control and curcumin microemulsion-treated group at a dose of 30 mg/kg were normal with normal glomerulus and normal tubules (Figure 8A,B). The kidney micrograph of thioacetamide-treated rats with a dose of 30 mg/kg showed extensive bleeding (Figure 8C). Treatment with curcumin microemulsions reduced thioacetamide-intoxicated histological changes in kidneys (Figure 8D).

## 4. Conclusions

The F127-based microemulsions synthesized in this work and loaded with curcumin showed high encapsulation efficiency and prolonged release of the drug. In vitro studies revealed that cancer cells were more sensitive to curcumin microemulsions than normal human cells. In vivo assessments indicated protective effects against thioacetamide-induced oxidative stress and hepatic injury in rats. Overall, the microemulsions prepared in this study represent a potentially effective drug delivery system for curcumin in advanced anticancer therapies.

## Figures and Tables

**Table 1:** Serum biochemical parameters and lipid peroxidation in rats. *, ** and *** indicate a statistically significant difference with *p* < 0.05, *p* < 0.01, and *p* < 0.001, respectively, for the control group.

Item	Treatment			
	Control	Curcumin Microemulsion 30 mg/kg	Thioacetamide 100 mg/kg	Thioacetamide 100 mg/kg + Curcumin 30 mg/kg
AST (U/L)	78.0 ± 8.6	66.3 ± 10.0	96.7 * ± 15.0	77.8 ± 11.7
ALT (U/L)	31.1 ± 4.7	36.2 ± 4.1	48.3 ** ± 9.6	41.2 * ± 8.1
MDA (nmol/mL)	137.9 ± 20.8	130.6 ± 21.0	164.0 * ± 21.5	141.7 ± 13.1
BUN (mg/dL)	14.3 ± 2.8	13.8 ± 2.0	24.2 *** ± 4.5	15.2 ± 1.6
Creatinine (mg/dL)	0.95 ± 0.17	1.1± 0.21	2.01 *** ± 0.39	1.20 ± 0.48
Liver SOD (U/g protein)	18.0 ± 2.3	26.0 ** ± 8.0	11.7* ± 2.2	16.5 ± 2.4
Liver CAT (U/g protein)	52.5 ± 8.4	62.9 * ± 79.9	38.1 ** ± 7.4	44.5 ± 6.8

## Data Availability

Data are included within this article.

## Supplementary Materials

The following are available online at https://www.mdpi.com/2079-4991/11/3/817/s1, Figure S1: The optimized structures of compounds studied in the present work, Figure S2: The initial (left) and final (right) structures of the different studied configurations and the related interaction energy (in kJ/mol), Figure S3: The molecular graph of the CURF1 complex obtained from the DFT calculation, Figure S4: The distances between the H and O atoms at the sites of existence the intermolecular hydrogen bond for A: CURF2 and B: CURSodium3 complexes, Figure S5: Correlation between the calculated ∇^2^*ρ*(r) with the $E_{HB}^{*}$ energies, Figure S6: The HOMO and LUMO orbitals of the CURF2 complex at M06-2X/6-31G* method, Table S1: The selected topological parameters of investigated complexes (in a.u.) and the energies of the intermolecular hydrogen bond ($E_{HB}^{*}$ in kJ mol^−1^)) for all studied complexes, calculated at the M06-2X/6-31G* level, Table S2: The NBO analysis of all studied configurations, Table S3: The values of |${\mathrm{HOMO}}_{(monomer_{1}}$_)_-$\;{\mathrm{LUMO}}_{(monomer_{2}}$_)_| and the |${\mathrm{HOMO}}_{\left(monomer_{2}\right)}$-${\mathrm{LUMO}}_{\left(monomer_{1}\right)}$| energies at M06-2X levels, Table S4: Thermodynamic parameters (all in kJ/mol) of all studied complexes.
