# Supplementary material for: Biochemical, Ameliorative and Cytotoxic Effects of Newly Synthesized Curcumin Microemulsions: Evidence from In Vitro and In Vivo Studies
Source: Nanomaterials (Basel). 2021 Mar 23;11(3):817. doi: 10.3390/nano11030817 (PMC8004644; doi:10.3390/nano11030817)
Supplement: Supplementary file 1 [file nanomaterials-11-00817-s001.zip › Supplementary material/Invitro certificate.pdf]

Zahedan University Of Medical Sciences

Research Ethics Certificate

Approval ID:	IR.ZAUMS.REC.1399.517	Approval Date:	2021-03-01
Evaluated by:	Zahedan University Of Medical Sciences		
Status:	Approved		
Approval Statement:	The project was found to be in accordance to the ethical principles and the national norms and standards for conducting Medical Research in Iran.<br>Notice:<br>1. Although the proposal has been approved by the research ethics committee, meeting the professional and legal requirements is the sole responsibility of the PI and other project collaborators.<br>2. This certificate is reliant on the proposal/documents received by this committee on 2021-03-01. The committee must be notified by the PI as soon as the proposal/documents are modified.		
Proposal Title:	Toxicity/morphology assessment of the effects of synthesized magnetic nanoparticles, colloidal nanocarriers, drug-loaded nanoparticles, and carbon nanotubes on non-cancerous and cancerous human cell lines		
Principal Investigator:	Name: saman sargazi<br>Email: sgz.biomed@gmail.com		

Dr. Seyed Mohammad Hashemi Shahri
Director of University/Regional Research Ethics Committee
Zahedan University Of Medical Sciences

Dr. Noor Mohammad Bakhshani
Secretary of University/Regional Research Ethics Committee
Zahedan University Of Medical Sciences
