# Supplementary material for: Biochemical, Ameliorative and Cytotoxic Effects of Newly Synthesized Curcumin Microemulsions: Evidence from In Vitro and In Vivo Studies
Source: Nanomaterials (Basel). 2021 Mar 23;11(3):817. doi: 10.3390/nano11030817 (PMC8004644; doi:10.3390/nano11030817)
Supplement: Supplementary file 1 [file nanomaterials-11-00817-s001.zip › Supplementary material/Invivo certificate.pdf]

UOZ
University of Zabol

ETHICS COMMITTEE CERTIFICATE OF APPROVAL

This is to certify that

Project Title: Investigating on the effects of curcummin and doxorubicin-loaded colloidal nanocarriers on oxidative stress parameters in male Wistar rats.

Principal Researchers: Mohammad Amiri Sarkarizi, Dr Mohammad Reza Hajinezhad , Dr Abbas Rahdar

Dated: 3-march-2021

Meets the requirements of the Research Ethics Committee. Of University of Zabol

Ethical code: IR.UOZ.REC.1399.009

SIGNED

M. Erfanian

Secretary, Ethics Committee
