# Supplementary material for: Biochemical, Ameliorative and Cytotoxic Effects of Newly Synthesized Curcumin Microemulsions: Evidence from In Vitro and In Vivo Studies
Source: Nanomaterials (Basel). 2021 Mar 23;11(3):817. doi: 10.3390/nano11030817 (PMC8004644; doi:10.3390/nano11030817)
Supplement: Supplementary file 1 [file nanomaterials-11-00817-s001.zip › Supplementary material/Supplementary information.docx]

**Biochemical, ameliorative and cytotoxic effects of newly synthesized curcumin microemulsions: Evidence from *in vitro* and *in vivo* studies**

Abbas Rahdar^1,^*, Mohammad Reza Hajinezhad ^2^, Saman Sargazi ^3^, Maryam Zaboli ^4^, Mahmood Barani ^5^, Francesco Baino ^6,^*, Muhammad Bilal ^7^, Esmael Sanchooli ^8^

1. Department of Physics, University of Zabol, Zabol, P. O. Box. 98613-35856, Iran
2. Basic Veterinary Science Department, Veterinary Faculty, University of Zabol, Zabol, P. O. Box. 98613-35856, Iran, Email: hajinezhad@uoz.ac.ir
3. Cellular and molecule Research Center, Resistant Tuberculosis Institute, Zahedan University of Medical Sciences, Zahedan, Iran, Email: sgz.biomed@gmail.com
4. Department of chemistry, University of Birjand, Birjand, Iran, Email: Zaboli_maryam@yahoo.com
5. Department of Chemistry, Shahid Bahonar University of Kerman, Kerman, Iran, mahmoodbarani7@gmail.com
6. Institute of Materials Physics and Engineering, Applied Science and Technology Department, Politecnico di Torino, Corso Duca degli Abruzzi 24, 10129 Torino, Italy
7. School of Life Science and Food Engineering, Huaiyin Institute of Technology, Huaian 223003, China; [bilaluaf@hotmail.com](mailto:bilaluaf@hotmail.com)
8. Department of chemistry, University of Zabol, Zabol, P. O. Box. 98613-35856, Iran

Email: esmael.sanchooli@gmail.com

* Correspondence: [a.rahdar@uoz.ac.ir](mailto:a.rahdar@uoz.ac.ir) (A.R.), [francesco.baino@polito.it](mailto:francesco.baino@polito.it) (F.B.)


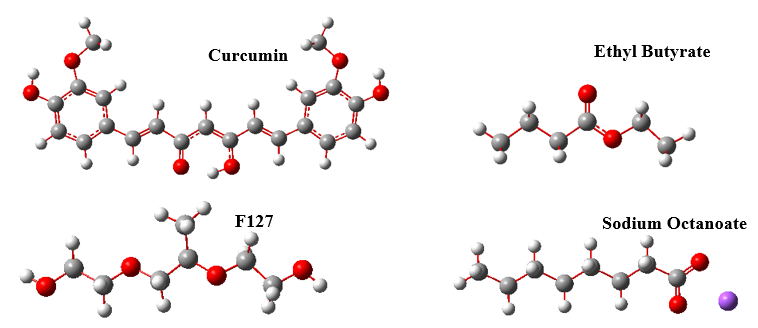


**Figure S1.** The optimized structures of compounds studied in the present work.


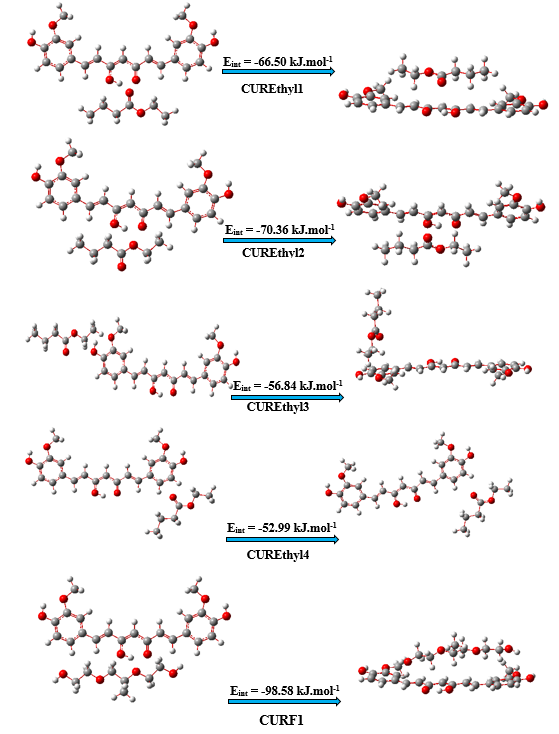


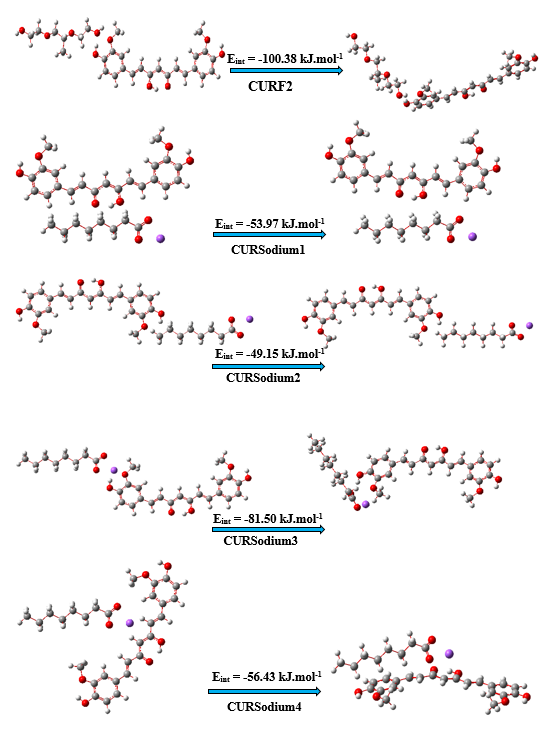


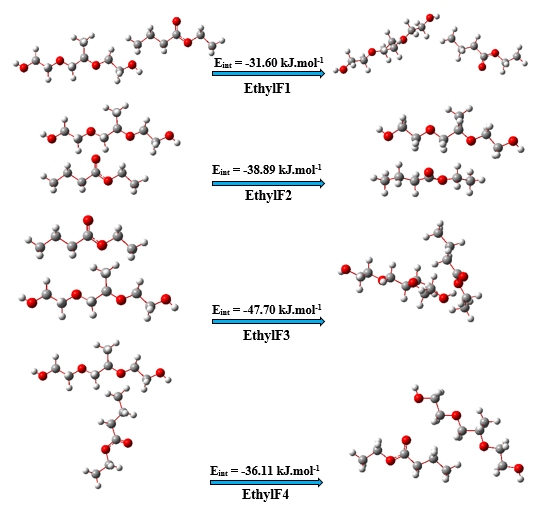


**Figure S2.** The initial (left) and final (right) structures of the different studied configurations and the related interaction energy (in kJ/mol).

**Table S1.** The selected topological parameters of investigated complexes (in a.u.) and the energies of the intermolecular hydrogen bond (E^*^_HB_ in kJmol^-1^) for all studied complexes, calculated at the M06-2X/6-31G* level.

| **CURF1** | H_30_-O_73_ | ^^H_30_-O_73_ | H(r) | E*_HB_ | **CUREthyl4** | H_38_-O_52_ | ^^H_38_-O_52_ | H(r) | E*_HB_ | **EthylF1** | H_40_-O_7_ | ^^H_40_-O_7_ | H(r) | E*_HB_ |
| --- | --- | --- | --- | --- | --- | --- | --- | --- | --- | --- | --- | --- | --- | --- |
|  | 0.0082 | 0.0311 | 0.0016 | -5.9162 |  | 0.0092 | 0.0361 | 0.0019 | -6.9793 |  | 0.0075 | 0.0284 | 0.0015 | -5.2818 |
|  | H_58_-O_9_ | ^^H_58_-O_9_ | H | E*_HB_ |  | H_39_-O_52_ | ^^H_39_-O_52_ | H | E*_HB_ | **EthylF2** | H_47_-O_9_ | ^^H_47_-O_9_ | H | E*_HB_ |
|  | 0.0069 | 0.0265 | 0.0015 | -4.7825 |  | 0.0099 | 0.0377 | 0.0018 | -7.5827 |  | 0.0050 | 0.0204 | 0.0013 | -3.3162 |
|  | H_53_-O_9_ | ^^H_53_-O_9_ | H | E*_HB_ | **CURSodium1** | H_71_-O_10_ | ^^H_71_-O_10_ | H | E*_HB_ |  | H_18_-O_32_ | ^^H_18_-O_32_ | H | E*_HB_ |
|  | 0.0051 | 0.0207 | 0.0013 | -3.4048 |  | 0.0142 | 0.0470 | 0.00152 | -11.4413 |  | 0.0061 | 0.0224 | 0.0013 | -3.9933 |
|  | H_53_-O_12_ | ^^H_53_-O_12_ | H | E*_HB_ |  | H_13_-O_35_ | ^^H_13_-O_35_ | H | E*_HB_ |  | H_22_-O_32_ | ^^H_22_-O_32_ | H | E*_HB_ |
|  | 0.0089 | 0.0313 | 0.0015 | -6.2926 |  | 0.0081 | 0.0316 | 0.00169 | -5.93071 |  | 0.0054 | 0.0202 | 0.0012 | -3.4818 |
| **CURF2** | H_49_-O_2_ | ^^H_49_-O_2_ | H | E*_HB_ |  | H_38_-O_20_ | ^^H_38_-O_20_ | H | E*_HB_ |  | H_26_-O_38_ | ^^H_26_-O_38_ | H | E*_HB_ |
|  | 0.0085 | 0.0343 | 0.0018 | -6.4192 |  | 0.0043 | 0.0175 | 0.00113 | -2.76341 |  | 0.0062 | 0.0239 | 0.0014 | -4.1878 |
|  | H_47_-O_54_ | ^^H_47_-O_54_ | H | E*_HB_ |  | H_16_-O_35_ | ^^H_16_-O_35_ | H | E*_HB_ | **EthylF3** | H_8_-O_32_ | ^^H_8_-O_32_ | H | E*_HB_ |
|  | 0.0414 | 0.1290 | -0.0035 | -51.4312 |  | 0.0081 | 0.0357 | 0.00203 | -6.38852 |  | 0.0243 | 0.0860 | 0.0013 | -24.8117 |
|  | H_52_-O_27_ | ^^H_52_-O_27_ | H | E*_HB_ | **CURSodium2** | H_24_-O_28_ | ^^H_24_-O_28_ | H | E*_HB_ |  | H_5_-O_33_ | ^^H_5_-O_33_ | H | E*_HB_ |
|  | 0.0068 | 0.0262 | 0.0015 | -4.7059 |  | 0.0098 | 0.0327 | 0.0014 | -6.9353 |  | 0.0115 | 0.0398 | 0.0016 | -8.7748 |
| **CUREthyl1** | H_63_-O_12_ | ^^H_63_-O_12_ | H | E*_HB_ |  | H_23_-O_53_ | ^^H_23_-O_53_ | H | E*_HB_ |  | H_13_-O_33_ | ^^H_13_-O_33_ | H | E*_HB_ |
|  | 0.0092 | 0.0322 | 0.0015 | -6.5515 |  | 0.0064 | 0.0252 | 0.0015 | -4.4325 |  | 0.0055 | 0.0213 | 0.0013 | -3.6327 |
|  | H_57_-O_9_ | ^^H_57_-O_9_ | H | E*_HB_ | **CURSodium3** | H_67_-O_10_ | ^^H_67_-O_10_ | H | E*_HB_ | **EthylF4** | H_22_-O_32_ | ^^H_22_-O_32_ | H | E*_HB_ |
|  | 0.0067 | 0.0256 | 0.0014 | -4.5945 |  | 0.0138 | 0.0472 | 0.00164 | -11.1842 |  | 0.0039 | 0.0160 | 0.0011 | -2.4798 |
| **CUREthyl2** | H_67_-O_12_ | ^^H_67_-O_12_ | H | E*_HB_ |  | H_66_-O_9_ | ^^H_66_-O_9_ | H | E*_HB_ |  | H_40_-O_9_ | ^^H_40_-O_9_ | H | E*_HB_ |
|  | 0.0108 | 0.0373 | 0.0016 | -8.0610 |  | 0.0509 | 0.1619 | -0.00662 | -70.4928 |  | 0.0052 | 0.0200 | 0.0012 | -3.3882 |
|  | H_57_-O_9_ | ^^H_57_-O_9_ | H | E*_HB_ |  | H_15_-O_45_ | ^^H_15_-O_45_ | H | E*_HB_ |  |  |  |  |  |
|  | 0.0077 | 0.0284 | 0.0015 | -5.3908 |  | 0.0062 | 0.0248 | 0.00147 | -4.29086 |  |  |  |  |  |
| **CUREthyl3** | H_62_-O_2_ | ^^H_64_-O_2_ | H | E*_HB_ | **CURSodium4** | H_62_-O_9_ | ^^H_62_-O_9_ | H | E*_HB_ |  |  |  |  |  |
|  | 0.0085 | 0.0343 | 0.0018 | -6.4192 |  | 0.0072 | 0.0278 | 0.00154 | -5.08131 |  |  |  |  |  |
|  | H_65_-O_2_ | ^^H_65_-O_2_ | H | E*_HB_ |  | H_22_-O_47_ | ^^H_22_-O_47_ | H | E*_HB_ |  |  |  |  |  |
|  | 0.0076 | 0.0292 | 0.0016 | -5.3885 |  | 0.0045 | 0.0175 | 0.0011 | -2.84709 |  |  |  |  |  |
|  | H_63_-O_27_ | ^^H_63_-O_27_ | H | E*_HB_ |  | H_23_-O_45_ | ^^H_23_-O_45_ | H | E*_HB_ |  |  |  |  |  |
|  | 0.0070 | 0.0265 | 0.0015 | -4.8238 |  | 0.0076 | 0.0283 | 0.00151 | -5.32085 |  |  |  |  |  |


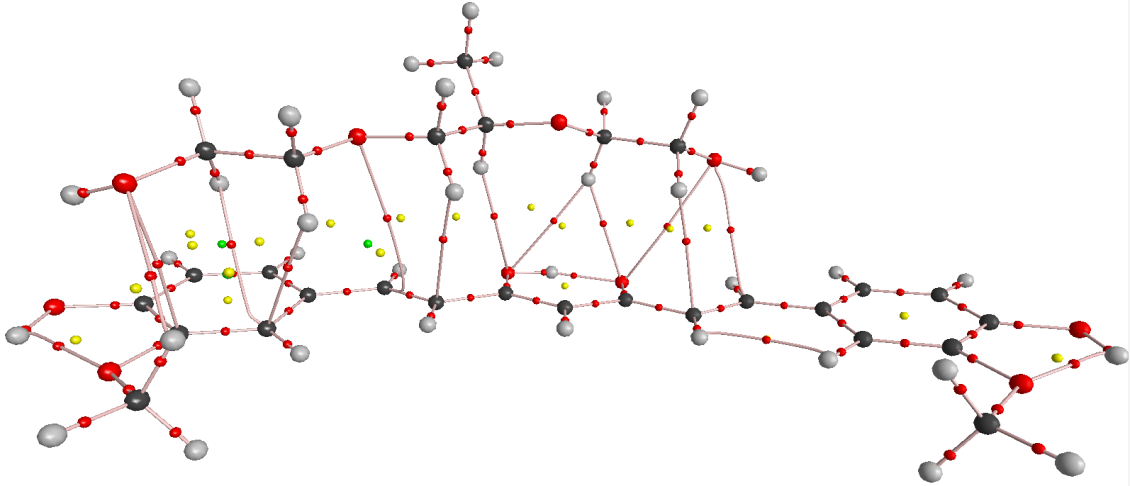


**Figure S3.** The molecular graph of the CURF1 complex obtained from the DFT calculation.


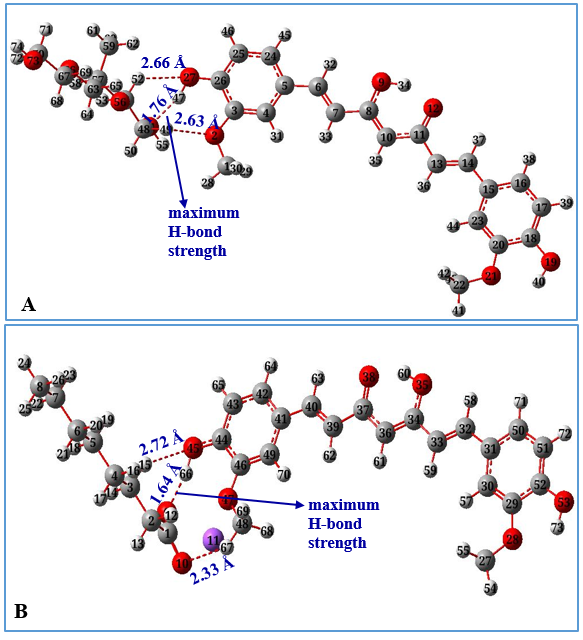


**Figure S4.** The distances between the H and O atoms at the sites of existence the intermolecular hydrogen bond for A: CURF2 and B: CURSodium3 complexes.

**Figure S5.** Correlation between the calculated ∇^2^ρ(r) with the $E_{HB}^{*}$ energies.

**The NBO analysis**

NBO analysis was utilized to understand and describe the charge delocalization within the molecule. Table S2 shows the second-order perturbation energy (E^(2)^) values obtained from the NBO analysis. The NBO analysis shows that the transfer of electrons occurs from the orbitals of F127, ethyl butyrate, and sodium octanoate to the anti-bond orbital of curcumin molecule in the CURF, CUREthyl, and CURSodium complexes. Also, in the EthylF complex, the direction of the charge transfer is from the lone pair (Lp) of the oxygen atom of F127 to the sigma antibonding orbital (*σ**) of ethyl butyrate. In the positions with intermolecular hydrogen bonds, charge transfer is from the proton acceptor to the proton donor.

**Table S2.**  The NBO analysis of all studied configurations.

| CURF1 | Lp_(O73)_ → *_(C1-H30)_ | CURSodium2 | _(C7-H22)_ → *_(O53-H73)_ |
| --- | --- | --- | --- |
|  | 0.61 |  | 0.29 |
| CURF2 | Lp_(O54)_ → *_(O27-H47)_ | **CURSodium3** | Lp_(O9)_ → *_(O45-H66)_ |
|  | 23.26 |  | 19.67 |
| CUREthyl1 | Lp_(O53)_ → *_(C11-O12)_ | **CURSodium5** | Lp_(O9)_ → *_(C39-C40)_ |
|  | 0.32 |  | 0.35 |
| CUREthyl2 | _(C54-H63)_ → *_(C11-O12)_ | **EthylF1** | Lp_(O7)_ → *_(C30-C31)_ |
|  | 0.36 |  | 0.47 |
| CUREthyl3 | _(C54-H63)_ → *_(C25-C26)_ | **EthylF2** | Lp_(O26)_ → *_(C30-H38)_ |
|  | 0.15 |  | 0.75 |
| CUREthyl4 | Lp_(O52)_ → *_(C16-H38)_ | **EthylF3** | Lp_(O9)_ → *_(C29-H36)_ |
|  | 0.82 |  | 1.58 |
| CURSodium1 | Lp_(O10)_ → *_(C50-H71)_ | **EthylF4** | Lp_(O9)_ → *_(C31-H40)_ |
|  | 4.19 |  | 0.80 |

HOMO–LUMO analyses

Close inspection of Figure S6 shows that the HOMO is distributed on the F127, and the LUMO orbital is positioned on the curcumin molecule in the complex. This result indicates that the transfer of electron density has happened between the HOMO of F127 and the LUMO of curcumin. On the other hand, the partial charge transfer will happen in partnership with a mixture of the filled orbital of one unit and the vacant orbital of another.


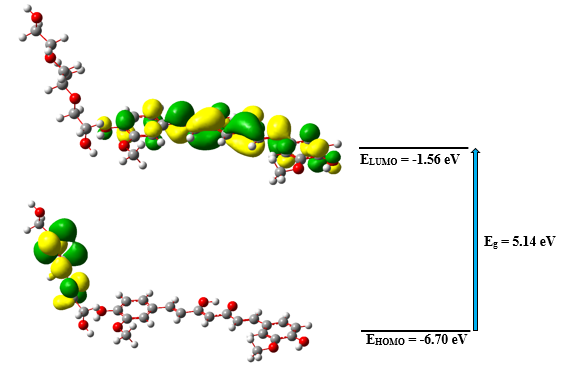


**Figure S6.** The HOMO and LUMO orbitals of the CURF2 complex at M06-2X/6-31G* method.

**Table S3**. The values of |$\mathrm{HOMO}_{({monomer}_{1}}$_)_-$\mathrm{LUMO}_{{(monomer}_{2}}$_)_| and the |$\mathrm{HOMO}_{{(monomer}_{2})}$-$\mathrm{LUMO}_{({monomer}_{1})}$| energies at M06-2X levels.

| \|HOMO_(Curcumin)_-LUMO_(F127)_\| | \|HOMO_(F127)_-LUMO_(Curcumin)_\| |
| --- | --- |
| 0.36 | **0.27** |
| \|HOMO_(Curcumin)_-LUMO_(Ethyl Butyrate)_\| | \|HOMO_(Ethyl Butyrate)_-LUMO_(Curcumin)_\| |
| 0.31 | **0.29** |
| \|HOMO_(Curcumin)_-LUMO_(Sodium Octanoate)_\| | \|HOMO_(Sodium Octanoate)_-LUMO_(Curcumin)_\| |
| 0.25 | **0.24** |
| \|HOMO_(Ethyl Butyrate)_-LUMO_(F127)_\| | \|HOMO_(F127)_-LUMO_(Ethyl Butyrate)_\| |
| 0.46 | **0.39** |

Table S3 shows that in the CURF, CUREthyl, and CURSodium complexes, the electron density can be transferred from the HOMO of F127, ethyl butyrate, and sodium octanoate to the LUMO of curcumin. Also, in the EthylF complex, the direction of the electron density transfer is between the HOMO of F127 and LUMO of ethyl butyrate. This result is consistent with the obtained results of NBO analyses.

**Table S4.** Thermodynamic parameters (all in kJ/mol) of all studied complexes.

| Complexes | ∆H | ∆G | T∆S |
| --- | --- | --- | --- |
| CURF1 | -51.000 | 16.240 | -67.240 |
| CURF2 | -34.659 | 15.094 | -49.753 |
| CUREthyl1 | -35.268 | 21.479 | -56.748 |
| CUREthyl2 | -36.660 | 21.461 | -58.121 |
| CUREthyl3 | -17.733 | 44.880 | -62.613 |
| CUREthyl4 | -5.149 | 29.540 | -34.688 |
| CURSodium1 | -14.569 | 20.250 | -34.819 |
| CURSodium2 | -1.686 | 34.840 | -36.526 |
| CURSodium3 | -32.230 | 19.330 | -51.560 |
| CURSodium4 | -30.458 | 19.253 | -49.711 |
| EthylF1 | -2.752 | 29.038 | -31.790 |
| EthylF2 | -21.043 | 27.904 | -48.947 |
| EthylF3 | -34.980 | 18.048 | -53.027 |
| EthylF4 | -8.554 | 30.773 | -39.327 |

The negative ΔH values show that the formation process of all complexes is exothermic and enthalpically favored. The T**∆**S value shows the entropy changes during the formation process of complexes. The high negative values of T**∆**S determine the positive **∆**G values. In other words, the formation processes of these systems are thermodynamically unfavorable; therefore, the probability of complex formation is controlled by the entropic factor and needs a larger entropy than the energy changes (which means that T**∆**S > **∆**H). It can be said that the complexes with lower **∆**G values are relatively more stable, whereas those with higher standard Gibbs energy of formation are unstable. Consequently, CURF1 and CURF2 complexes with lower **∆**G values are more stable than the other complexes.
